# Protective Effect of Strawberry Extract against Inflammatory Stress Induced in Human Dermal Fibroblasts

**DOI:** 10.3390/molecules22010164

**Published:** 2017-01-21

**Authors:** Massimiliano Gasparrini, Tamara Y. Forbes-Hernandez, Francesca Giampieri, Sadia Afrin, Bruno Mezzetti, Josè L. Quiles, Stefano Bompadre, Maurizio Battino

**Affiliations:** 1 Dipartimento di Scienze Cliniche Specialistiche ed Odontostomatologiche (DISCO)-Sez. Biochimica, Facoltà di Medicina, Università Politecnica delle Marche, Via Ranieri 65, 60100 Ancona, Italy; m.gasparrini@univpm.it (M.G.); tamara.forbe@gmail.com (T.Y.F.-H.); f.giampieri@univpm.it (F.G.); dolla.bihs@gmail.com (S.A.); 2 Area de Nutrición y Salud, Universidad Internacional Iberoamericana (UNINI), Campeche C.P. 24040, Mexico; 3 Dipartimento di Scienze Agrarie, Alimentari e Ambientali, Università Politecnica delle Marche, 60131 Ancona, Italy; b.mezzetti@univpm.it; 4 Department of Physiology, Institute of Nutrition and Food Technology ‘‘José Mataix”, Biomedical Research Centre, University of Granada, Granada, C.P. 18000, Spain; jlquiles@ugr.es; 5 Dipartimento di Scienze Biomediche e Sanità Pubblica, Facoltà di Medicina, Università Politecnica delle Marche, Via Ranieri 65, 60131 Ancona, Italy; s.bompadre@univpm.it; 6 Centre for Nutrition & Health, Universidad Europea del Atlantico (UEA), 39011 Santander, Spain

**Keywords:** strawberry, LPS, oxidative stress, inflammation, prevention

## Abstract

A protracted pro-inflammatory state is a major contributing factor in the development, progression and complication of the most common chronic pathologies. Fruit and vegetables represent the main sources of dietary antioxidants and their consumption can be considered an efficient tool to counteract inflammatory states. In this context an evaluation of the protective effects of strawberry extracts on inflammatory stress induced by *E. coli* LPS on human dermal fibroblast cells was performed in terms of viability assays, ROS and nitrite production and biomarkers of oxidative damage of the main biological macromolecules. The results demonstrated that strawberry extracts exerted an anti-inflammatory effect on LPS-treated cells, through an increase in cell viability, and the reduction of ROS and nitrite levels, and lipid, protein and DNA damage. This work showed for the first time the potential health benefits of strawberry extract against inflammatory and oxidative stress in LPS-treated human dermal fibroblast cells.

## 1. Introduction

A common denominator in the pathogenesis of most chronic diseases is the involvement of oxidative stress, connected to an unbalanced equilibrium between oxidant production and antioxidant defenses [1]. The excessive production of reactive oxygen species (ROS), no longer adequately controlled by antioxidant defense systems, can lead to a wide range of effects in both human physiological and pathological conditions, affecting different cellular processes, such as proliferation, metabolism, differentiation, and survival, antioxidant and anti-inflammatory response, tissue injury and cell death by activating apoptosis and necrosis processes [2,3]. Moreover the inflammatory condition related to oxidative stress plays an important role also in the process of skin wound healing [4,5,6] which represents a complex process regulated by a large variety of different growth factors, cytokines and hormones and which involves various cellular and extracellular matrix components. At the cellular level the main targets of ROS attack are lipids, proteins and DNA [7]. Lipid damage occurs mainly in cellular membranes, which are especially vulnerable to peroxidation due to their contents in polyunsaturated fatty acids. Proteins can undergo direct and indirect damage following interaction with ROS, as well as peroxidation, damage to specific aminoacid residues, changes in their tertiary structure, degradation, and fragmentation. Finally, ROS can interact also with DNA causing several types of damage, such as loss of purines and modification of DNA bases, single- and double-DNA breaks, DNA-protein cross-linkage, and damage to the DNA repair system and to the deoxyribose moieties [8,9]. In this context dietary antioxidants from fruit and vegetables fill an important beneficial role and clinical and epidemiological data from literature corroborate this hypothesis [10,11,12]. Focusing on fruits, in recent decades, specific subgroups of fruits have been taken into account, to facilitate the observation and promote their specific health benefits [13]. Among these, the strawberry represents a relevant source of folate, is rich in vitamin C and contains various phytochemicals and bioactive compounds, which confer to this berry antioxidant, anticancer, anti-neurodegenerative and anti-inflammatory properties, as indicated in recent in vitro and in vivo studies [11,14,15,16,17]. In this context, the aim of the present work was to evaluate the effects of methanolic extracts from “Alba” strawberry cultivar on the inflammatory status induced by *E. coli* lipopolysaccharide (LPS) on the human dermal fibroblasts (HDF) cell line. The endotoxin LPS constitutes an outer membrane structure and an important virulence factor of the cell wall of Gram-negative bacteria, commonly used as an inflammatory agent [18,19]. HDF represent a standard cell model for different kinds of toxicity determinations [20,21]; it is also known that HDF recruit immune cells via soluble mediators and contribute to the progression of inflammatory processes [22,23]. For this reason it is very important to better understand how fibroblasts produce and secrete different inflammatory mediators, in relation to LPS-induced stress. In our work, mainly the phytochemical and antioxidant characterization of Alba extract was detected. In order to assess the effect of strawberry treatment on HDF cells in the presence or absence of LPS, cell viability, intracellular ROS and nitrite (NO_2_^−^) production assays were performed and the biomarkers of oxidative damage to the principal biological macromolecules were estimated. To the best of our knowledge this is the first study which investigates the protective effect of strawberry extract on LPS-induced damage in HDF cell line.

## 2. Results and Discussion

### 2.1. Strawberry Fruit Analysis

The extract from Alba strawberry cultivar showed a good content of polyphenols (TPC) (2.52 mg Gallic Acid Equivalent/g fresh weight (FW)), vitamin C (vit C) (0.58 mg vit C/g FW) and flavonoids (TFC) (0.66 mg Catechin Equivalent/g FW) (Table 1). Five anthocyanins (ACYs) pigments were detected through HPLC-MS/MS analysis, with pelargonidin (Pg) 3-glucoside (39.74 mg/100 g FW) and Pg 3-malonylglucoside (6.69 mg/100 g FW) being the most representative components (Table 1). Taking into account the folate profile, Alba cultivar contained 0.99 µg of folinic acid calcium salt hydrate/g FW and 0.06 µg of 5-methyltetrahydrofolic acid/g FW. These compounds represent, together with the other bioactive compounds present in strawberries, the elements responsible for several beneficial actions in human health as previously reported by different authors [24,25]. Finally, Alba extract also showed a high total antioxidant capacity (TAC) value, with 22.85, 22.64 and 7.71 μmol Trolox Equivalent/g FW for Ferric Reducing Antioxidant Power (FRAP), Trolox Equivalent Antioxidant Capacity (TEAC) and 2,2-DiPhenyl-1-PicrylHydrazyl (DPPH) respectively, confirming the results obtained for other strawberry varieties (Table 1) [26,27,28].

### 2.2. Strawberry and LPS Effects on Cell Viability

**Figure 1:**
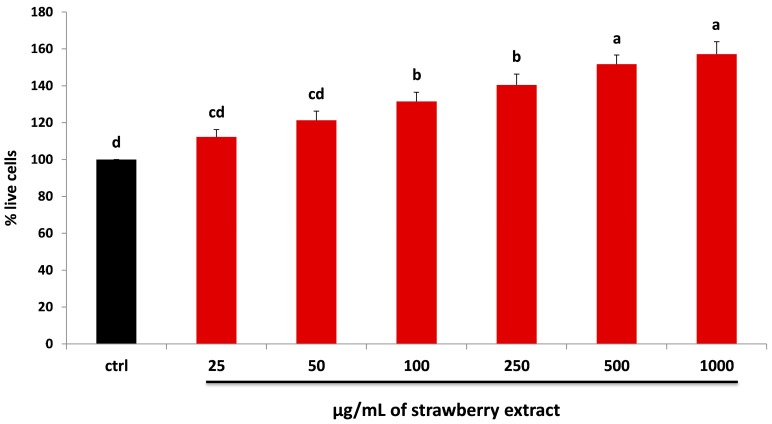
MTT assay for the determination of cell viability in HDF cells treated with different concentrations of strawberry extracts (25–1000 µg/mL) for 24 h (red bars). Black bar represents the control group. Data are expressed as mean values ± standard deviation (SD). Values with different superscript letters are significantly different (*p* < 0.05).

First, HDF cells were incubated for 24 h with different concentrations of dried strawberry extracts in order to evaluate their possible cytotoxic effect. After the treatment, an increase in cell viability was observed in a dose-dependent manner, up to +57% with 1000 µg/mL of strawberry extract (*p* < 0.05) (Figure 1). These results are in line with previous studies which highlighted the anti-cytotoxic effect of strawberry treatment on cellular viability [26,27,29].

**Figure 2:**
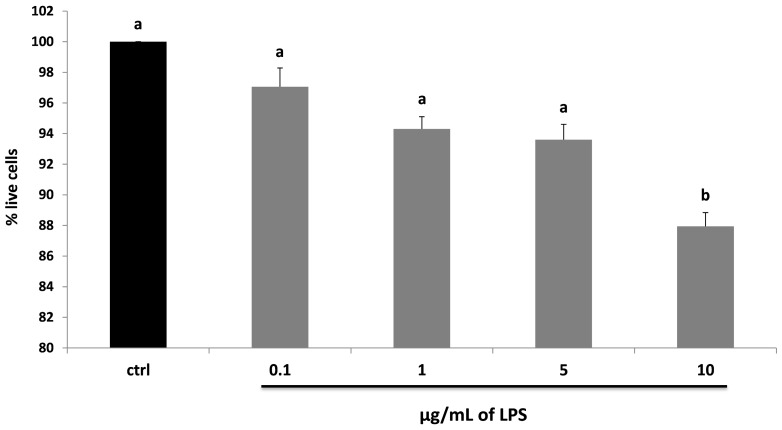
MTT assay for the determination of cell viability in HDF cells treated with different concentrations of LPS (0.1–10 µg/mL) for 24 h (grey bars). Black bar represents the control group. Data are expressed as mean values ± SD. Values with different superscript letters are significantly different (*p* < 0.05).

To test the effect of LPS on cell viability, HDF were treated with different endotoxin concentrations for 24 h. Only 10 μg/mL of LPS produced a significant reduction on viability, −13% compared to control (*p* < 0.05), while all the other doses did not produce any statistical difference compared to untreated cells (Figure 2).

**Figure 3:**
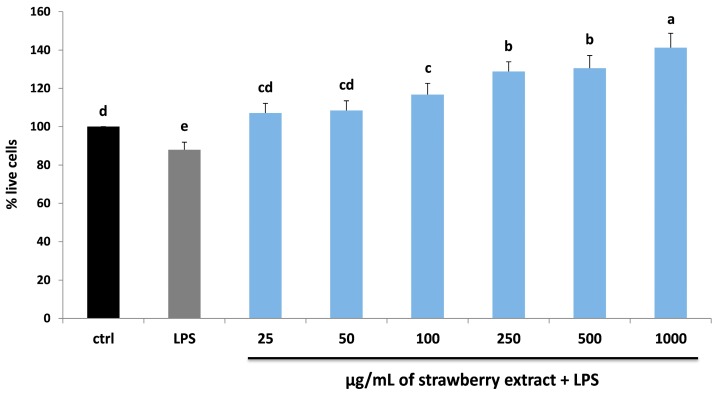
MTT assay for the determination of cell viability in HDF cells treated with LPS (10 µg/mL) for 24 h (grey bar) and different concentrations of strawberry extract (25–1000 µg/mL) for 24 h and then with LPS (blue bars). Black bar represents the control group. Data are expressed as mean values ± SD. Values with different superscript letters are significantly different (*p* < 0.05).

Finally, the effect of strawberry supplementation before the LPS treatment on HDF cell viability was also evaluated, in order to highlight the possible protective role of strawberry pre-treatment against LPS damage. As shown in Figure 3, strawberry extracts showed a protective role at all the different concentrations applied, producing a significant increase in cell viability compared to LPS-treated cells already at 100 μg/mL (*p* < 0.05). These preliminary data permitted to obtain a first observation of the concentration range of strawberry extracts and LPS, used for the following analysis.

### 2.3. Strawberry Treatment Reduced ROS Intracellular Production

**Figure 4:**
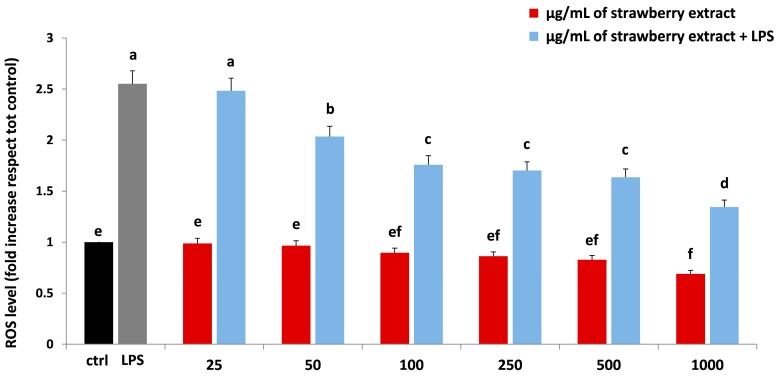
ROS level in HDF cells treated with different concentrations of strawberry extracts (25–1000 µg/mL) for 24 h (red bars), LPS (10 µg/mL) for 24 h (grey bar) and with different concentrations of strawberry extracts and then with LPS (blue bars). Black bar represents the control group. Data are expressed as mean values ± SD. Columns with different superscript letters are significantly different (*p* < 0.05).

The measure of ROS intracellular production could represent a very useful parameter to quantify the oxidative stress induced by LPS. Endogenous or exogenous inflammatory stimuli induced the generation of ROS, resulting in hyperactivation of inflammatory response, tissue damage and oxidative stress phenomena [30]. In our work, the protective effect of strawberry extract on LPS-induced ROS production was demonstrated in a dose-dependent manner (Figure 4). In HDF cells, strawberry treatment showed a reduction of ROS amount compared to the control group, which became significant at 1000 µg/mL (*p* < 0.05). A significant reduction in the amount of ROS compared to the LPS treatment was obtained with a strawberry concentration of 50 μg/mL (*p* < 0.05), 100 μg/mL (*p* < 0.05) and 1000 µg/mL (*p* < 0.05). For this reason these doses of strawberry were used for all further analysis.

The results obtained for the first time with strawberries in HDF were in line with those found by several authors, who tested the efficacy of different bioactive compounds against LPS-induced damage in different cellular models, such as human gingival fibroblasts and macrophages [31,32,33].

### 2.4. Strawberry Treatment Decreased NO_2_^−^ Accumulation

**Figure 5:**
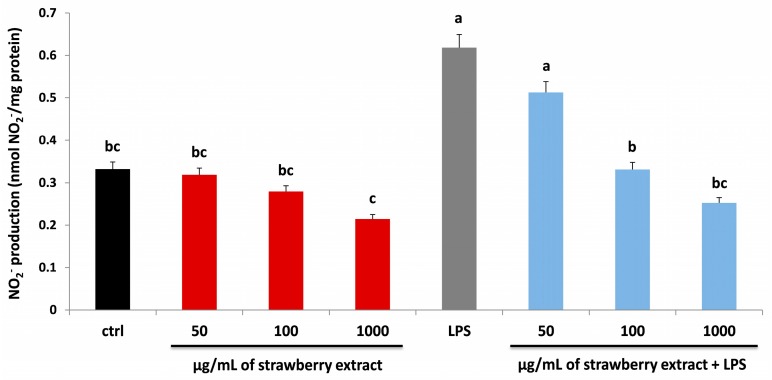
NO_2_^−^ production in HDF cells treated with different concentrations of strawberry extracts (50, 100, 1000 µg/mL) for 24 h (red bars), LPS (10 µg/mL) for 24 h (grey bar) and with different concentrations of strawberry extracts and then with LPS (blue bars). Black bar represents the control group. Data are expressed as mean values ± SD. Columns with different superscript letters are significantly different (*p* < 0.05).

LPS can lead to the release of pro-inflammatory cytokines, activating a second level of inflammatory cascades including lipid mediators, cytokines and adhesion molecules such as nitric oxide (NO) [34]. NO is an important effector and regulatory molecule with different biological functions and it is involved in many physiological and pathophysiological processes. As shown in Figure 5, strawberry extract was able to reduce the level of NO derivative nitrite production compared to untreated HDF cells, significantly at 1000 µg/mL (*p* < 0.05). On the contrary, LPS-treatment significantly increased (*p* < 0.05) the NO_2_^−^ level, which was efficiently counteracted with strawberry pre-treatment in a dose-dependent manner, restoring values similar to the control group with extract at 100 µg/mL.

Although few studies have so far been carried out to investigate the involvement of NO_2_^−^ production in HDF cells after strawberry/LPS treatment, our results are in line with previous data collected on different cell models (murine fibroblast cell line and macrophages) [32,33,35].

### 2.5. Strawberry Treatment Reduced Protein, Lipid and DNA Damage

In order to determine the level of protein and lipid oxidative damage, common markers of protein and lipid oxidation were evaluated, as shown in previous works [36,37,38]. As can be seen in Table 2, strawberry treatments ameliorated protein carbonyl content and increased glutathione (GSH) levels compared to untreated cells, while HDF subjected to LPS treatment showed considerable protein damage (*p* < 0.05), as highlighted by the highest value of carbonyl content and the lowest value of GSH. Pre-treatment with strawberry extracts improved the levels of LPS-induced protein damage: values statistically similar to the control groups were obtained, both for GSH and carbonyl groups, with the extract at 100 μg/mL. Similar results were obtained in relation to lipid oxidation marker (Table 2). Our results demonstrated that strawberry extracts significantly reduced the thiobarbituric acid-reactive substances (TBARS) level compared to the control group (*p* < 0.05); moreover Alba pre-treatment efficiently counteracted the LPS-oxidative effect, restoring values similar to untreated cells at 100 μg/mL.

**Figure 6:**
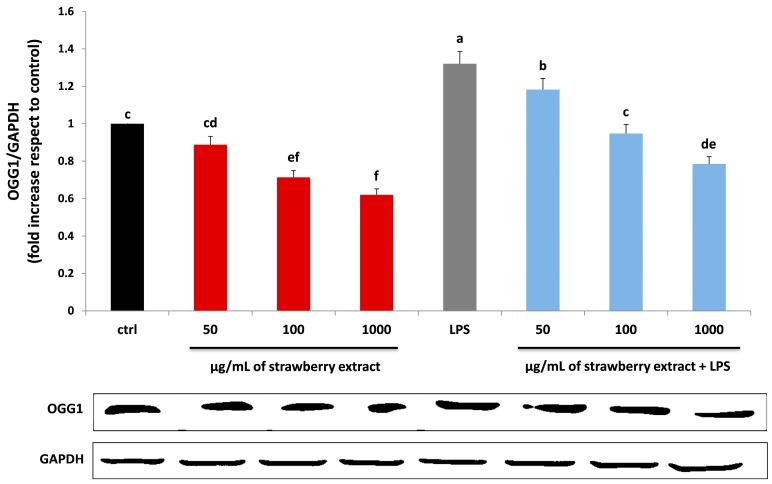
Level of protein related to DNA damage (OGG1) in HDF cells treated with different concentrations of strawberry extracts (50, 100, 1000 µg/mL) for 24 h (red bars), LPS (10 µg/mL) for 24 h (grey bar) and with different concentrations of strawberry extracts and then with LPS (blue bars). Black bar represent the control group. Data are expressed as mean values ± SD. Columns with different superscript letters are significantly different (*p* < 0.05).

The DNA damage level was also investigated, through the evaluation of 8-Oxoguanine glycosylase (OGG1) expression. A variety of enzymes able to repair oxidant-induced DNA modifications are contained in mitochondrion and nucleus, which represent the two major targets of oxidative stress. DNA damage occurs when the endogenous antioxidant network and DNA repair systems are flooded [39]. The expression level of 8-Oxo-7,8-dihydro-2-deoxyguanosine reflects the rate of oxoguanosine formation in DNA, which is a symptom of oxidative stress. HDF treatment with strawberry extracts reduced OGG1 protein expression in a dose-dependent manner. On the contrary LPS, significantly increased the level of OGG1 (*p* < 0.05), which was efficiently reduced by pre-incubation with strawberries, already at 100 μg/mL (Figure 6).

## 3. Materials and Methods 

### 3.1. Chemicals and Reagents

All chemicals and solvent were of analytical grade. Cyanidin (Cy)-3-glucoside, Pelargonidin (Pg)-3-glucoside, ferrous sulphate (FeSO_4_), 2,20-azino-bis-(3-ethylbenzothiazoline-6-sulfonic acid) diammonium salt (ABTS), 2,2-diphenyl-1-picrylhydrazyl (DPPH)6-hydroxy-2,5,7,8-tetramethyl-chroman-2-carboxylic acid (Trolox), Griess reagent (catalog number G4410), dinitrophenylhydrazine, 5,5 dithiobis (2-nitrobenzoic acid), thiobarbituric acid (TBA), RIPA buffer (product number R 0278), LPS (*Escherichia coli* serotype 055:B5, product number L2880) and all other reagents and solvents were purchased from Sigma-Aldrich chemicals, Milan, Italy. CellROX^®^ Orange reagent were purchased from Invitrogen^TM^, Life Technologies, Milan, Italy. Dulbecco’s Modified Eagle Medium (DMEM), were obtained from Carlo Erba Reagents, Milan, Italy, as well as all other products for cell cultivation. Primary and secondary antibodies were purchased from Santa Cruz Biotechnology, Dallas, TX, USA and Bioss Inc., Woburn, MA, USA and all the other products for western blot analysis from Bio-Rad Laboratories, Inc., Hercules, CA, USA.

### 3.2. Strawberry Fruit Preparation and Analysis

Ripe fruits of Alba strawberry cultivar were harvested from plants grown at the Azienda Agraria Didattico Sperimentale “P. Rosati” in Agugliano (Ancona, Italy). Within 2 h after harvest, whole fruits were stored at −20 °C. To make the strawberry extract, frozen strawberries were submitted to methanolic extraction as previously described [27]. Briefly, 10 g of fruits were homogenized in 100 mL of the 80% methanol aqueous solution acidified with 0.1% formic acid using an Ultra-Turrax T25 homogeniser (Janke & Kunkel, IKA Labortechnik) and then stirred for 2 h at 4 °C in the dark. The extract was centrifuged at 3500 rpm for 15 min twice sequentially in order to sediment solids, and the supernatant was filtered through a 0.45 mm Minisart filter (PBI International), transferred to 5 mL amber glass vials and stored at −20 °C prior to analysis. Folin-Ciocalteu method [40] and the aluminium chloride spectrophotometric method [41] were used to determine TPC and TFC, respectively. Vit C and folate contents were analysed by HPLC system [42,43], while ACYs solid-phase extraction and HPLC-MS/MS analysis were performed as previously described [44]. Finally, TAC was determined using TEAC [45], FRAP assays [46] and the DPPH free radical method [47]. Results were expressed as mean value of three replicates ± SD.

### 3.3. Cell Culture and Strawberry/LPS Treatments

HDF were Human Dermal Fibroblasts isolated from adult skin provided by GIBCO^®^ Invitrogen cell. HDF were plated into a T-75 flasks and cultured as previously described [48]. Cells were maintained in HeraCell CO_2_ incubator at 37 °C with 5% CO_2_ and the medium was changed every 2–3 days. All the tests and the different pellet preparations were conducted on cells between the 4th and 6th passage. Strawberry extract was concentrated under vacuum to eliminate total methanol and resuspended in Dulbecco’s Modified Eagle Medium (DMEM). Cells were treated with extract of Alba cultivar and LPS at different concentrations/times according to the assay performed.

### 3.4. Cell Viability Assay

Cell viability was determined using the MTT assay as previously described [49], with some modifications. The assay represents a quick and comprehensive colorimetric method based on the ability of viable cells to reduce a soluble tetrazolium salt, 3-(4,5-dimethylthiazol-2-yl)-2, 5-diphenytetrazolium bromide to blue formazan crystals [50]. Briefly, HDF were seeded into 96-well plates at a density of 5 × 10^3^ cells/well and left to adhere for 16-18 h. After the cells were adherent, they were incubated with: (i) DMEM only for the control group; (ii) dried strawberry extract at 25, 50, 100, 250, 500 and 1000 μg/mL for 24 h; (iii) LPS at 0.1, 1, 5 and 10 μg/mL for 24 h; (iv) dried strawberry extract for 24 h and then with LPS at 10 μg/mL for 24 h. The concentration values and the exposure time applied for dried strawberry and LPS treatments were chosen according to previous cytotoxicity studies (data not shown). At the end of the different incubations (i, ii, iii, iv) fibroblasts were washed twice with PBS and incubated with a salt solution of MTT at a concentration of 0.5 mg/mL for 2 h at 37 °C. The medium was then removed and the crystals were dissolved in DMSO. The level of colored formazan derivative was analysed on a microplate reader (ThermoScientific Multiskan EX) at a wavelength of 590 nm. Cell viability was expressed as a percentage of live cells compared to control. The data reported represent average values from three independent experiments.

### 3.5. TALI^®^ ROS Concentration Assay 

The determination of intracellular ROS levels was performed using the probe CellROX^®^ Orange reagent according to the manufacturer’s instructions. Briefly, on the first day of the assay 1.5 × 10^5^ cells were seeded in a 6-well plate, and left to adhere for 16–18 h. The cells were treated, the day after seeding, with: (i) DMEM only for the control group; (ii) dried strawberry extract at 25, 50, 100, 250, 500 and 1000 μg/mL for 24 h; (iii) LPS at 10 μg/mL for 24 h; (iv) dried strawberry extract for 24 h and then with LPS for 24 h. The concentration of LPS at 10 μg/mL was chosen according to previous cell viability results. At the end of each treatment (i, ii, iii, iv), the medium was removed and collected, then CellROX^®^ Orange Reagent was added (1:500 dilution) to 1 mL of DMEM. Samples were incubated at 37 °C for 30 min, centrifuged at 320 g and then resuspended in phosphate-buffered saline solution (PBS). After labeling with CellROX^®^ Orange Reagent, cells were analyzed with the Tali^®^ Image-Based cytometer (Invitrogen^TM^, Life Techonoliges, Milan, Italy). Control cells were used to determine baseline levels of ROS and to set the fluorescence threshold for the instrument. Each treatment was performed in three replicates and the final results were reported as fold increase compared to control.

### 3.6. Determination of Nitrite Production

NO_2_^−^ accumulation in cell culture media was determined by Griess method [51]. Briefly, 1 × 10^6^ cells were seeded in a T75 flask, and left to adhere overnight. The day after seeding, HDF were subjected to different treatments: (i) DMEM only for the control group; (ii) dried strawberry extract at 50, 100 and 1000 μg/mL for 24 h; (iii) LPS at 10 μg/mL for 24 h; (iv) dried strawberry extract for 24 h and then with LPS for 24 h. The concentration values for dried strawberry treatments were chosen according to previous ROS production results. At the end of the diverse incubations (i, ii, iii, iv), the different cell supernatants were collected, the samples (1 mL) were mixed with equal volume of Griess reagent (1 mL of 1:1 0.1% naphthyl-ethylenediamine and 1% sulfanilamide in 5% phosphoric acid) in a tube, and incubated in the dark for 10 min at room temperature. The absorbance of the reaction mixture was measured at 540 nm on a microplate reader (ThermoScientific Multiskan EX). The concentration of NO_2_^−^ in the sample was determined using a standard curve realized with sodium nitrite (NaNO_2_) in a working range of 0.1–6.25 µM. Each treatment was carried out in three replicates and the final results were expressed as nmol NO_2_^−^/mg protein.

### 3.7. Measurements of the Protein and Lipid Oxidative Damage

For the measurement of protein and lipid oxidative damage, HDF were treated as explained for nitrite evaluation. At the end of the different incubations (i, ii, iii, iv), HDF were treated with RIPA buffer, incubated on ice for 5 minutes and the lysate obtained was stored at −80 °C until analyses [52]. In cellular lysates, protein oxidation was detected through the measurement of protein carbonyl content and GSH levels were determined by the dinitrophenylhydrazine method [53] and 5,5 dithiobis (2-nitrobenzoic acid) assay [54], respectively. Lipid peroxidation was performed by the assay of TBARS production, according to a standardized method [55]. In the case of carbonyl content and GSH the results were expressed as nmol/mg prot, while for TBARS level as nmol/100 mg prot. Each sample was analyzed in three replicates.

### 3.8. Immunoblotting Assay for DNA Damage Evaluation

For immunoblotting assays, HDF were treated as explained for nitrite evaluation. At the end of the different incubations (i, ii, iii, iv), HDF were collected, washed with PBS, lysed in 100 μL lysis buffer (120 mmol/L NaCl, 40 mmol/L Tris [pH 8], 0.1% NP40) containing protease and phosphatase inhibitor cocktails (Roche Diagnostics, Mannheim, Germany) and centrifuged for 15 min at 13,000 g. Proteins from cell supernatants were then charged on a 10% dodecyl sulfate-polyacrylamide running gel (Bio-Rad, Hercules, CA, USA), and at the end of the electrophoresis run, proteins were transferred to nitrocellulose membranes, using a Trans-Blot Turbo Transfer System (Bio-Rad Laboratories, Inc., Hercules, CA, USA). After that the membranes were blocked with TBS-T containing 5% non-fat milk for 1 h at room temperature. OGG1 antibody was used to detect protein by Western Blotting. GAPDH protein was used for the measurement of the amount of protein analysed. Membranes were incubated overnight at 4 °C with the primary antibodies solution, (1:500 diluted *v*/*v*) and then probed for 1 h at room temperature with their specific HRP-conjugated secondary antibodies (1:80,000 diluition *v*/*v*). Proteins were visualized using a chemiluminescence method (C-DiGit Blot Scanner, LI-COR, Bad Homburg, Germany). Quantification of gene expression was made using the software provided by the manufacturer of the Blot Scanner (Image Studio 3.1) and data were expressed as fold increase compared to control. The assay was performed in three replicates. Protein amount was determined by the Bradford method [56].

### 3.9. Statistical Analysis

Statistical analyses were performed using STATISTICA software (Statsoft Inc., Tulsa, OK, USA). Data were subjected to one-way ANOVA analysis of variance for mean comparison, and significant differences among different treatments were calculated according to HSD Tukey’s multiple range test. Data are reported as mean ± SD. Differences at *p* < 0.05 were considered statistically significant.

## 4. Conclusions

Our results showed for the first time a clear and a dose-dependent protective effect of strawberry extract on LPS-induced damage in HDF cell line, improving cell viability, reducing ROS and NO_2_^−^ level and counteracting oxidative damage to the principal biological macromolecules. These preliminary results represent an interesting starting point: additional in vitro and in vivo studies will be necessary to identify the molecular pathways involved in the strawberry-mediated anti-inflammatory response and their mechanisms of action. Moreover further studies will be needed in order to detect the main classes of flavonoid compounds by evaluating and comparing the anti-inflammatory effect of anthocyanin and ellagitanin fractions compared to the whole extract.

## Figures and Tables

**Table 1:** Phytochemical and antioxidant capacity of Alba strawberry extract. Data are presented as mean value ± SD.

Parameter	Quantification
TPC (mg GAEq/g FW)	2.52 ± 0.01
vit C (mg vit C/g FW)	0.58 ± 0.02
TFC (mg CEq/g FW)	0.66 ± 0.01
ACYs (mg/100g FW)	
Cy-3-glucoside	3.11 ± 0.03
Pg 3-glucoside	39.74 ± 0.13
Pg 3-rutinoside	3.87 ± 0.16
Pg 3-malonylglucoside	6.69 ± 0.04
Pg 3-acetylglucoside	0.39 ± 0.01
Folate (µg folate/g FW)	
folinic acid calcium salt hydrate	0.99 ± 0.09
5-methyltetrahydrofolic acid	0.06 ± 0.01
TAC (µmol Teq/g FW)	
FRAP	22.85 ± 0.39
TEAC	22.64 ± 0.49
DPPH	7.71 ± 0.32

**Table 2:** Protein and lipid oxidation markers (protein carbonyl content, GSH and TBARS levels) in HDF cells treated with different concentrations of strawberry extracts (50, 100, 1000 µg/mL) for 24 h, LPS (10 µg/mL) for 24 h and with different concentrations of strawberry extracts and then with LPS. Columns belonging to the same set of data with different superscript letters are significantly different (*p* < 0.05).

Treatment	Protein Carbonyl Content (nmol/mg Prot)	GSH Level (nmol/mg Prot)	TBARS Level (nmol/100 mg Prot)
No treatment (ctrl)	65.54 ± 1.63 ^c,d^	25.89 ± 1.29 ^c,d^	21.02 ± 2.12 ^b,c^
Strawberry 50 µg/mL	54.97 ± 4.92 ^d,e^	26.18 ± 1.31 ^c,d^	18.12 ± 1.39 ^c,d^
Strawberry 100 µg/mL	41.15 ± 8.89 ^e,f^	37.63 ± 1.88 ^b^	14.56 ± 1.54 ^d,e^
Strawberry 1000 µg/mL	31.55 ± 7.28 ^f^	42.84 ± 2.14 ^a^	11.23 ± 1.47 ^e^
LPS	94.78 ± 4.74 ^a^	9.72 ± 0.49 ^f^	33.25 ± 2.89 ^a^
Strawberry 50 µg/mL + LPS	93.28 ± 4.66 ^a^	14.86 ± 0.74 ^e^	31.66 ± 3.54 ^a^
Strawberry 100 µg/mL + LPS	74.56 ± 3.72 ^b,c^	22.42 ± 1.12 ^d^	23.85 ± 1.58 ^b^
Strawberry 1000 µg/mL + LPS	62.24 ± 3.11 ^c,d^	31.89 ± 1.59 ^b,c^	16.23 ± 1.77 ^d^
